# A Behavioral and Electrophysiological Investigation of Effects of Visual Congruence on Olfactory Sensitivity During Habituation to Prolonged Odors

**DOI:** 10.1093/chemse/bjaa065

**Published:** 2020-10-09

**Authors:** Nicholas Fallon, Timo Giesbrecht, Anna Thomas, Andrej Stancak

**Affiliations:** 1 Department of Psychological Sciences, Institute of Psychology, Health, and Society, University of Liverpool, Eleanor Rathbone Building, Bedford Street South, Liverpool, UK; 2 Unilever Research and Development, Quarry Rd E, Wirral, UK

**Keywords:** cross-modal, desensitization, EEG, multisensory integration, olfaction

## Abstract

Congruent visual cues augment sensitivity to brief olfactory presentations and habituation of odor perception is modulated by central-cognitive processing including context. However, it is not known whether habituation to odors could interact with cross-modal congruent stimuli. The present research investigated the effect of visual congruence on odor detection sensitivity during continuous odor exposures. We utilized a multimethod approach, including subjective behavioral responses and reaction times (RTs; study 1) and electroencephalography (EEG, study 2). Study 1: 25 participants received 2-min presentations of moderate-intensity floral odor delivered via olfactometer with congruent (flower) and incongruent (object) image presentations. Participants indicated odor perception after each image. Detection sensitivity and RTs were analyzed in epochs covering the period of habituation. Study 2: 25 new participants underwent EEG recordings during 145-s blocks of odor presentations with congruent or incongruent images. Participants passively observed images and intermittently rated the perceived intensity of odor. Event-related potential analysis was utilized to evaluate brain processing related to odor–visual pairs across the period of habituation. Odor detection sensitivity and RTs were improved by congruent visual cues. Results highlighted a diminishing influence of visual congruence on odor detection sensitivity as habituation occurred. Event-related potential analysis revealed an effect of congruency on electrophysiological processing in the N400 component. This was only evident in early periods of odor exposure when perception was strong. For the first time, this demonstrates the modulation of central processing of odor–visual pairs by habituation. Frontal negativity (N400) responses encode the aspects of cross-modal congruence for odor–vision cross-modal tasks.

## Introduction

Integration of cross-modal sensory information in the brain is a dynamic, ongoing process that is subject to confounding top-down and bottom-up influences that affect the individual’s overall perception. Despite this, few studies investigate the interaction of olfactory–visual stimuli and, to our knowledge, none have considered whether typical confounding factors, such as habituation to odor, would affect this interaction. Whilst olfaction and vision operate via anatomically distinct brain pathways, both essentially serve the same function of object identification (Gottfried 2010). Research suggests a bidirectional relationship between vision and olfaction. Visual stimuli can facilitate odor detection (Gottfried and Dolan 2003) and identification (Dematte et al. 2009). Olfaction also influences fundamental aspects of visual processing, for example, binocular rivalry studies (which present a different visual stimuli concurrently to each eye) show visual dominance occurring for the lateralized image matching the presence of a congruent, compared to incongruent, odor (Zhou et al. 2010). The precise mechanisms underlying the integration of olfactory and visual information in the brain are not fully understood but may facilitate the effects of context and other top-down psychological influences (Robinson et al. 2015) or subjective experience (Amsellem et al. 2018).

Odor habituation describes the central-cognitive processes, such as changes in brain and behavioral responsiveness or sensitivity to odor, which occur during prolonged periods of exposure (Dalton 2000). Neuroimaging research with functional magnetic resonance imaging (fMRI) indicates that habituation is encoded in primary olfactory, including piriform, entorhinal cortex, and amygdale, and higher-order brain regions, such as anterior insula and hippocampus (Poellinger et al. 2001). Understanding whether cross-modal cues would interact with the process of habituation has relevance for the scientific understanding of the interaction between these processes and also for commercial applications where long-lasting influence of fragrance is often desirable. Appropriate visual cues could affect olfactory processing during habituation by redirecting attentional resources. Electroencephalography (EEG) studies have shown that focused attention increases olfactory event-related potentials (ERPs; Pause et al. 1997; Krauel et al. 1998; Geisler and Murphy 2000; Masago et al. 2001), and fMRI studies report modulations of brain activation responses when attention is focused toward an odor (Sabri et al. 2005; Zelano et al. 2005; Plailly et al. 2008; Veldhuizen and Small 2011). Research from our group has also previously highlighted the influence of endogenous attention on the process of habituation (Fallon et al. 2018).

Traditionally, multisensory integration was thought to occur in higher-order integrative brain processing regions, but more recent evidence suggests that at least some aspects are represented in primary sensory brain regions and directly affect perception (Meyer et al. 2011; Liang et al. 2013). Animal models using in vivo extracellular recordings from the olfactory tubercle have demonstrated the interaction between olfactory and auditory processing in this primary olfactory cortex (Wesson and Wilson 2010). fMRI studies have demonstrated an interaction effect for activation in orbitofrontal, inferior parietal lobule, and posterior cingulate cortices during cross-modal odor–visual processing (Gottfried et al. 2004). However, to our knowledge, no research exists to consider if the effects of cross-modal interactions in primary or secondary olfactory cortices could be affected by olfactory habituation.

Previously, EEG recordings were analyzed using ERP analysis aligned to the onset of images to study the effects of odors on visual processing (Lorig et al. 1993, 1995; Grigor 1995; Grigor et al. 1999; Bensafi et al. 2002; Castle et al. 2000; Robinson et al. 2015). These studies generally utilized a variation of an oddball paradigm, with a common–rare split of congruent and incongruent odor–visual pairs. The paradigm relies on the premise of olfactory priming, wherein the odor precludes the arrival of the visual stimuli and influences brain activity in a manner that leads to some quantifiable modulation of subsequent visual processing (Bensafi et al. 2002). We can index this modulation of visual processing using ERP analysis of EEG data.

In previous research, the most common waveform modulated by odor–visual congruence is the N400, a negative deflection in frontal electrodes occurring from 250 to 500 ms after the onset of visual stimuli, which was previously proposed to encode the degree of congruence between an olfactory prime stimulus and the visual target (Bensafi et al. 2002). A recent review of N400 research concludes that it incorporates aspects of perception, attention, memory, and semantics (Kutas and Federmeier 2011). The amplitude of this N400 wave was increased for incongruous and rare odor–visual pairs (Grigor 1995; Grigor et al. 1999), and modulation of the N400 wave has been demonstrated by studies using both pleasant (Sarfarazi et al. 1999) and unpleasant odor–visual pairs (Castle et al. 2000). Research from our lab previously identified the modulation of the N400 component during affective face perception with hedonically congruent or incongruent odor priming (Cook et al. 2017). Together, this evidence suggests that odors may influence a late, semantic stage of visual processing as previously proposed (Grigor et al. 1999; Sarfarazi et al. 1999), although one recent study did not identify N400 differences and instead pointed toward an influence of odors on early (N1) visual processing (Robinson et al. 2015). It should be noted that few, if any, cross-modal EEG studies have focused on odor detection outcomes.

All previous research of olfactory–visual interaction utilized short bursts of odor with long interstimulus intervals to prevent habituation. Therefore, it is not known whether the effects of odor–visual congruence influence the process of olfactory habituation. Furthermore, the brain mechanisms which govern the interaction of odor–visual processing, and how these fluctuate during perceptual changes during prolonged odor exposure, are not known. In this research, we first investigated the influence of congruent visual cues on olfactory performance during a period of prolonged odor exposure to induce olfactory habituation. To determine whether habituation modulated central processing of olfactory–visual pairs, we analyzed neural responses to congruent and incongruent visual stimuli across a period of prolonged odor using ERP analysis and distributed source localization analysis of EEG. We hypothesized that congruent visual cues would lead to improved odor detection sensitivity, but that this improvement would reduce due to the process of olfactory habituation during a prolonged exposure. Furthermore, we expected that late-semantic components of processing for odor–visual pairs would be differentially affected by effects of cross-modal congruence and that this effect would be modulated as habituation occurred.

## Methods

### Participants

For study 1, 25 participants (12 males) aged 24.2 ± 3.62 years (mean ± standard deviation [SD]) were recruited. A separate cohort of 25 participants (13 males) aged 23.2 ± 3.99 years (mean ± SD) took part in study 2. In both cases, participants were recruited through digital and campus advertisements at the University of Liverpool. Written informed consent was obtained from all participants in accordance with the Declaration of Helsinki. The studies were approved by the University of Liverpool Research Ethics Committee. Participants aged between 18 and 35 years were considered for participation, and volunteers taking regular medication or those suffering from respiratory, neurological, or olfactory disease or disorders (according to self-report) were excluded. Eligibility and sense of smell were assessed prior to the experiment using the identification test from the “Sniffing Sticks” odor test battery (Hummel et al. 1997). In this test, participants were asked to identify 12 odors from 4 visually presented options, and a minimum score of 9 correct probes was required for inclusion in either study. All volunteers were compensated for time and travel expenses.

### Odor stimuli

For both studies the floral-green fragrance “New Day” (Unilever Ltd) was utilized at 5% concentrations diluted in propylene glycol (1,2-Propanediol 95%, Sigma-Aldrich Co.). This concentration was found to be perceived as moderate intensity following testing of a range of possible concentrations in psychophysical prestudies (Unilever Ltd, unpublished).

## Study 1

### Procedure

**Figure 1.**
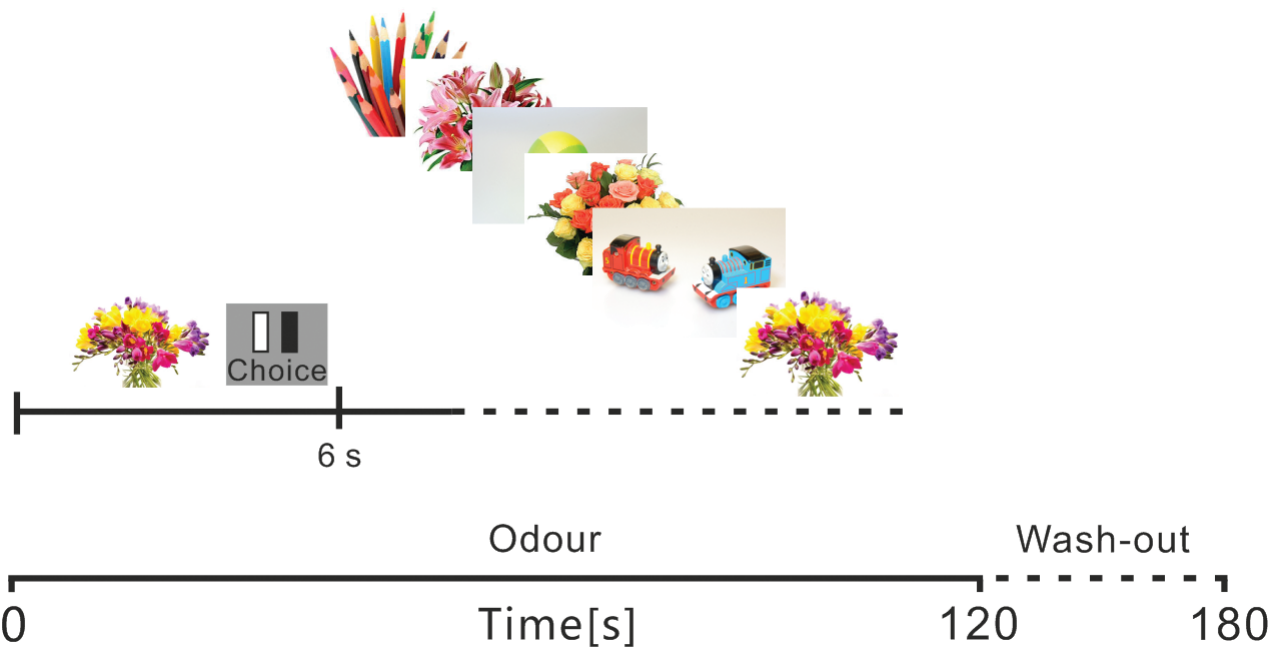
Flowchart of an example block from study 1. Each odor block contained 20 (6 s) trials comprising a rest cross (1.5 s), picture presentation (0.5 s), blank screen (1 s), and a response period (3 s).

Participants attended the EEG laboratory in the Department of Psychological Sciences at the University of Liverpool. Participants were seated 1 m from a 19-inch computer monitor and a PneumoTrace II Piezo-electric transducer was fitted around the torso at the level of the epigastrium to record respiratory movements (ADInstruments Pty Ltd). The experiment consists of 10 blocks of prolonged (120 s) odor exposure at a flow rate of 2.2 L/min; there was a 1-min rest period between blocks when participants were exposed to a constant flow of clean air, which was passed through pure propylene glycol solution with a matching flow rate. Each block consisted of 20 trials that lasted for 6 s each and consisted of a rest cross (1.5 s) followed by a picture presentation (0.5 s), blank screen (1 s), and a rating period (3 s). Figure 1 shows the timeline of one experimental block. Each block contained 10 congruent picture trials (flowers in a variety of arrangements on a white background) and 10 incongruent stimuli (everyday objects on a white background). Each picture appeared twice in the experiment and the order of pictures was randomized in each block but conditions were alternated to maintain an even spread of each congruent and incongruent trials throughout the period of odor exposure. During the rating period, participants were required to click either the left or right mouse button to indicate whether they detected any odor during the previous picture. Participants were informed that they may or may not smell an odor at any time during the experiment and that they should simply indicate whether odor was present at that specific time. They were also instructed to give their response as quickly as possible. The lateralization of the mouse button corresponding to detection was counterbalanced across participants.

The olfactometer utilized was custom made, with 8 individual flow valves each benefitting from variable flow rates and a carbon-filtered air intake (OL-2, Dancer design Ltd). Odors were delivered via fluorinated ethylene propylene tubing of 2 mm diameter extending 2 cm below the nostrils. During the experiment, the ambient air in the chamber was constantly cleansed of residual odor using a carbon-filtered Blueair 203 Heppasilent Particle Filter system (Blueair AB).

### Odor detection and RT analysis

Bad trials (where neither option was selected during the 3-s response period) were removed for each participant; these represented less than 1% of total trials. Response data were divided into 5 time windows, each of which represented 24 s of odor presentations to evaluate whether the influence of congruent and incongruent stimuli differed across the period of odor exposure. Odor detection sensitivity for each picture condition and time window was calculated as the percentage of trials in each block when participants correctly detected the presence of odor. Mean RT for accurate and inaccurate responses was calculated in each subject for each time window and condition following the removal of improbable response trials (RT >1.5 s or RT <0.2 s). Two-way within-subjects ANOVA analysis for odor detection and RT was performed in SPSS v.21 (SPSS Inc.) to investigate the effects of congruence (picture type; congruent–incongruent) and time (5 time periods of 24 s covering the 2 min of exposure). Post hoc *t*-tests were utilized to investigate significant interaction effects and a 95% confidence level was employed throughout.

## Study 2

### Procedure

The second study also occurred in the EEG laboratory in the Department of Psychological Sciences at the University of Liverpool. Olfactometer and respiratory monitoring setup were identical to study 1. EEG was recorded continuously using a 129-channel Geodesics EEG System (Phillips-Electrical Geodesics Inc.) with the sponge-based HydroCel Geodesic Sensor Net (HCGSN-128) with vertex reference. The sensor net was aligned with respect to 3 anatomical landmarks: 2 preauricular points and the nasion. The electrode-to-skin impedances were kept below 50 kW and the recording bandpass filter was 0.01−200 Hz. The sampling rate was 1000 Hz.

**Figure 2.**
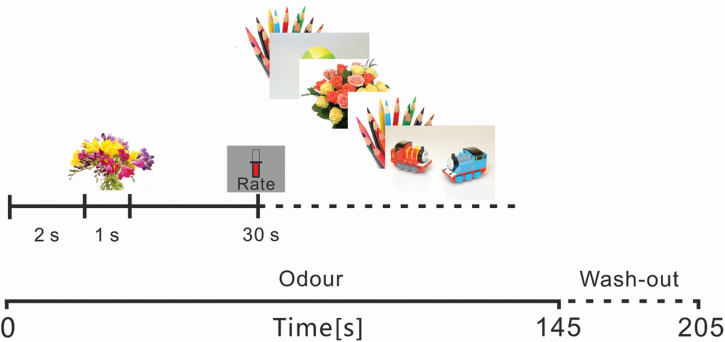
Flowchart of an example block from study 2. Each block of odor or clean air block contained 40 trials that lasted for 3 s consisting of blank screen (2 s) followed by a picture presentation (1 s) with 30 incongruent and 10 congruent trials. Participants rated odor intensity at the beginning of each block and every 30 s thereafter.

The second experimental paradigm consisted of 15 blocks; 10 utilized prolonged (145 s) odor exposure at a flow rate of 2.2 L/min and 5 blocks consisting of a continuous (145 s) flow of clean air at the same flow rate. Odorless blocks were interspersed evenly throughout the experiment with no 2 clean air blocks appearing consecutively (specifically in blocks 3, 5, 8, 10, and 13). Order of blocks was consistent across participants. Again, a 1-min clean air rest period was utilized between blocks. Each block consisted of 40 trials, which lasted for 3 s each consisting of a blank screen (2 s) followed by a picture presentation (1 s). The ratio of trials was skewed so that each block contained 30 object picture trials (expanded library from previous study) and 10 flower stimuli (same as previous). Each picture appeared 3 times in the total experiment. The order of pictures was pseudo-randomized in each block, with parameters to ensure that both conditions were dispersed equally across the block to allow for analysis, including segmentation into time windows. At the beginning of each block and every 30 s thereafter, a rating scale appeared on screen (5 s) with a visual analog scale for participants to rate their perceived level of odor intensity at that moment using a mouse click. The scale anchors ranged from “No odor” to “Extremely Intense.” Figure 2 shows the timeline of one example experimental block.

### ERP analysis

EEG data were preprocessed using BESA v.6.0 (MEGIS). Data were spatially transformed into reference-free data using a common average reference method (Lehmann 1987) and downsampled to 256 Hz. Oculographic and electrocardiographic artifacts were removed using principal component analysis (PCA; Berg and Scherg 1994) in BESA v6.0 software. This is an interactive process where the user first manually identifies a prototypical eyeblink or electrocardiogram artifact complex in continuous data. This examplar complex is utilized in PCA to identify and remove all instances that match this pattern. No more than 2 artifact components (eye blink and electrocardiogram) were removed per participant. Topographic maps of each individual’s artefact components were visually inspected to confirm typical topography before removal was performed. Data were visually inspected for the presence of movement or muscle artifacts, and epochs contaminated with artifacts were manually excluded. The mean number of trials remaining following artifact correction was 72.1 ± 6.52 (mean ± SD) for flower pictures in odor condition (72% of total possible trials), 221.4 ± 18.8 for object pictures in odor condition (73%), 105.04 ± 12.14 (mean ± SD) for object pictures in clean air condition (70%), and 35.96 ± 4.67 for flower pictures in clean air condition (72%).

ERPs associated with the onset of each type of picture in each odor condition were exported for the interval ranging from −200 to 1000 ms relative to stimulus onset (307 time points). This epoch was selected for ERP analysis as this period was found to adequately cover peaks in global field power and butterfly plots corresponding to the early, mid-, and long-latency ERP components (Figure 5A). The baseline period was from −200 to 0 ms relative to the onset of the picture, and EEG data was bandpass filtered from 0.1 to 40 Hz. Finally, data were exported to Matlab v.8.10 (The Mathworks Inc.) for statistical analysis utilizing EEGlab toolbox (Delorme and Makeig 2004).

To investigate the impact of odor habituation on the processing of congruent or incongruent visual images, ERPs from each odor and picture-type condition were segmented into five 24-s time windows that covered the period of exposure. To identify electrodes and ERP components suitable for investigation, we employed a collapsed functional localizer method (Luck 2014) that utilizes averaged data collapsed across one or more experimental manipulations to identify spatio-temporal clusters of interest. Grand average ERPs representing all odor–picture pair conditions were initially divided into 5 levels corresponding to the 5 time windows covering the period of prolonged exposure. A nonparametric analysis was performed to investigate the main effects of 5 levels of exposure time on brain processing of odor–visual pairs across all 129 electrodes and at every time point of the ERP. This analysis was performed using the Fieldtrip toolbox, implemented in EEGlab, and utilized 2000 permutations to counter the multiple comparisons required for the investigation of spatio-temporal data (Maris and Oostenveld 2007). This analysis indicated a contiguous cluster of frontal electrodes that demonstrated a significant effect of exposure time in the period 250–400 ms after picture onset (corresponding to frontal N400). Mean EEG voltages from this cluster and time period were exported for each participant in each odor, picture, and time-window condition. N400 amplitudes were analyzed using a 2 × 2 (odor × congruence) within-subjects ANOVA in SPSS v.21 (SPSS Inc.) to investigate the main effects and interactions of odor and picture type in each time window. A 95% confidence level was employed throughout.

### Source reconstruction

Cortical sources of significant differences in ERPs were analyzed using standardized low-resolution electromagnetic analysis (sLORETA, (Pascual-Marqui 2002), implemented in LORETA v.200840-403 (www.keyinst.unizh.ch/loreta). sLORETA evaluates distributed electrical sources by smoothing the inverted images using a Laplacian smoothing operator to give cortical maps of electrical activity that show a good localization accuracy (Greenblatt *et al.* 2005; Sekihara *et al.* 2005). Source maps were computed in a grid of 6239 voxels sized 5 × 5 × 5 mm^3^, covering the entire cortical mantle. The sLORETA method was applied to localize the cortical sources contributing to the topographic configuration of ERP from any time window that demonstrated significant effects identified in scalp-level analyses. Grand average sLORETA maps were generated representing the strongest cortical sources associated with all conditions using 5000 randomizations and an arbitrary *T* threshold (*T* > 15) was implemented to restrict maps to distinct cortical structures indicative of strongest cortical sources of scalp ERPs. Then, sLORETA values from each of these sources and for each condition were exported using 10-mm-diameter spherical region of interest (ROI) centered on the peak value of each cluster. The extracted values for each ROI were utilized in a 2 × 2 (odor × picture type) within-subjects ANOVA to consider the effects of congruence on source-level activations in ERP components that demonstrated significance in scalp data.

## Results

### Study 1

**Figure 3.**
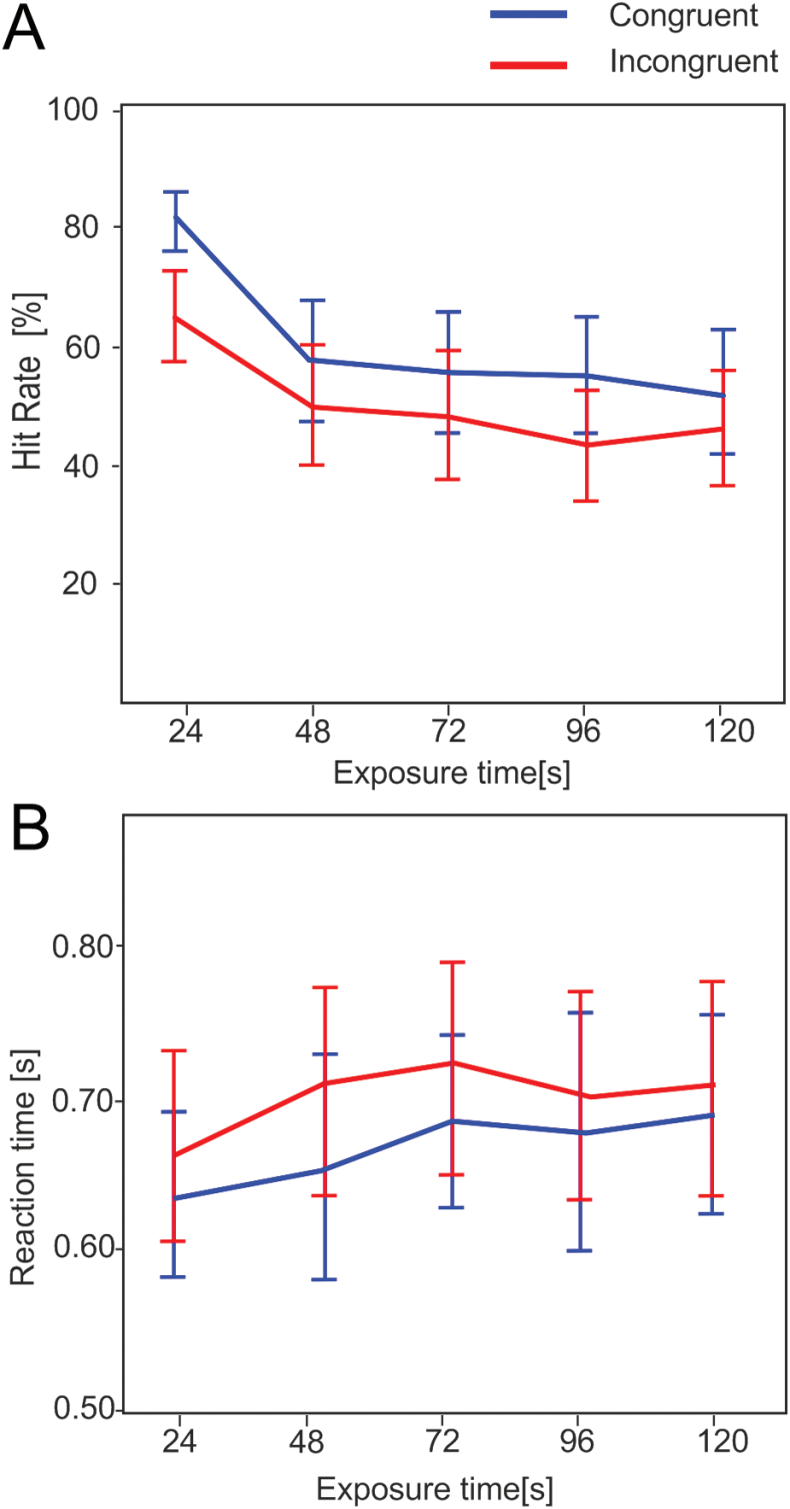
(A) Mean odor detection sensitivity (%) throughout the period of odor exposure and **(B)** mean RT (seconds) for correct responses to odor detection for congruent (blue) and incongruent (red) odor–visual pairs. Error bars illustrate 95% confidence intervals.

Subjective responses for perceived odor presence were recorded after each picture presentation, and mean odor detection sensitivity (%) was calculated for each subject in each of the 5 (24 s) time windows covering the period of odor exposure. A 2 × 5 (picture type × time) within-subjects ANOVA revealed a main effect of picture type (*F*(1,24) = 17.33, *P* < 0.001) with a greater proportion of accurate odor detections when flower pictures were presented. There was also a significant main effect of time (*F*(4,96) = 27.96, *P* < 0.001) with greater odor detection sensitivity evident in early time windows of the odor exposure. The standard interaction effect was not significant (*F*(4,96) = 2.38, *P* = 0.57), but the cubic interaction effect was highly significant (*F*(1,24) = 8.51, *P* = 0.008). This cubic polynomial represents an exponential model of change in odor detection sensitivity in congruent compared to incongruent conditions from early to later time windows of the odor exposure. Post hoc *t*-tests indicate that congruent images lead to more accurate odor detection, which is strongest in the first time window (*t*(24) = 6.44, *P* < 0.001), an effect that continues throughout the period of odor exposure until the difference is no longer significant in the final time window when odor intensity is lowest (*t*(24) = 1.1, *P* = 0.28). Figure 3A shows the mean sensitivity rate of detection for each time window.

RTs for correct odor detections following each type of picture presentation were exported and segmented into identical time windows. A 2 × 5(picture type × time) ANOVA for RTs revealed a significant effect of picture type (*F*(1,24) = 12.07, *P* = 0.002) demonstrating shorter RTs required to correctly identify the presence of odor in the congruent relative to incongruent condition. However, the main effect of time was not significant (*F*(4,92) = 2.20, *P* = 0.75), nor the interaction (*F*(4,92) = 0.24, *P* = 0.92). Figure 3B shows the mean RT for accurate responses in each time window of the odor exposure.

## Study 2

### Subjective intensity ratings

**Figure 4.**
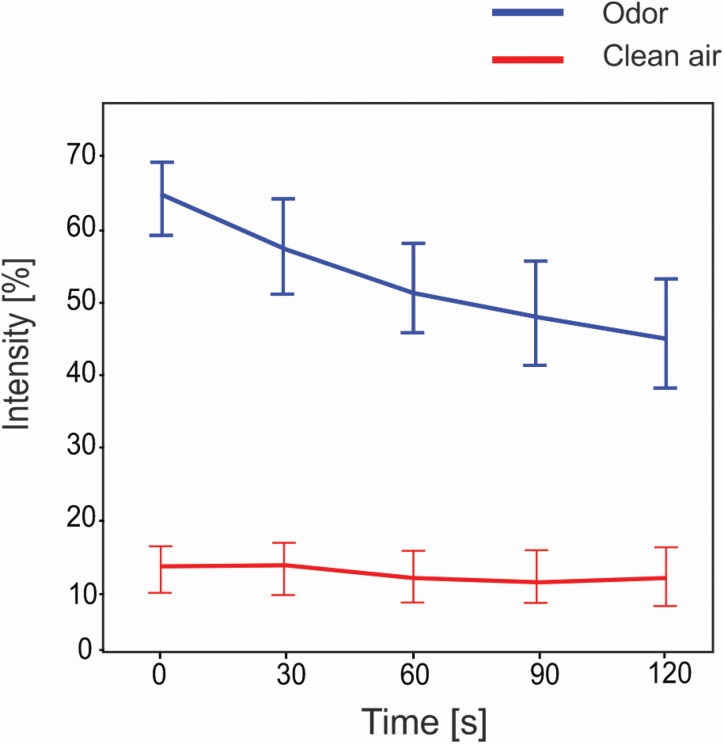
Mean subjective ratings of odor intensity throughout the period of exposure for clean air (red) and odor (blue) blocks. Error bars illustrate 95% confidence intervals.

Subjective ratings of perceived odor intensity were recorded in 30-s intervals throughout each block. A 2 × 5 (odor × time) within-subjects ANOVA revealed a main effect of odor, with greater intensity ratings reported across all time points when odor was present (*F*(1,24) = 169.47, *P* < 0.001). There was also a main effect of time, with higher odor ratings given at earlier time points in the exposure (*F*(1,24) = 30.80, *P* < 0.001). The interaction effect was also significant, indicating a difference in the effect of odor presence across different time periods (*F*(1,24) = 18.63, *P* < 0.001). Post hoc paired samples *t*-tests reveal a significant difference in perceived odor intensity between odor and clean air blocks at every time point as would be expected given the nature of the comparison. However, data indicate that the difference between odor conditions was greatest at the start of the presentations and decreased in a linear fashion indicative of habituation as the exposure prolonged (Figure 4).

### ERP analysis

**Figure 5.**
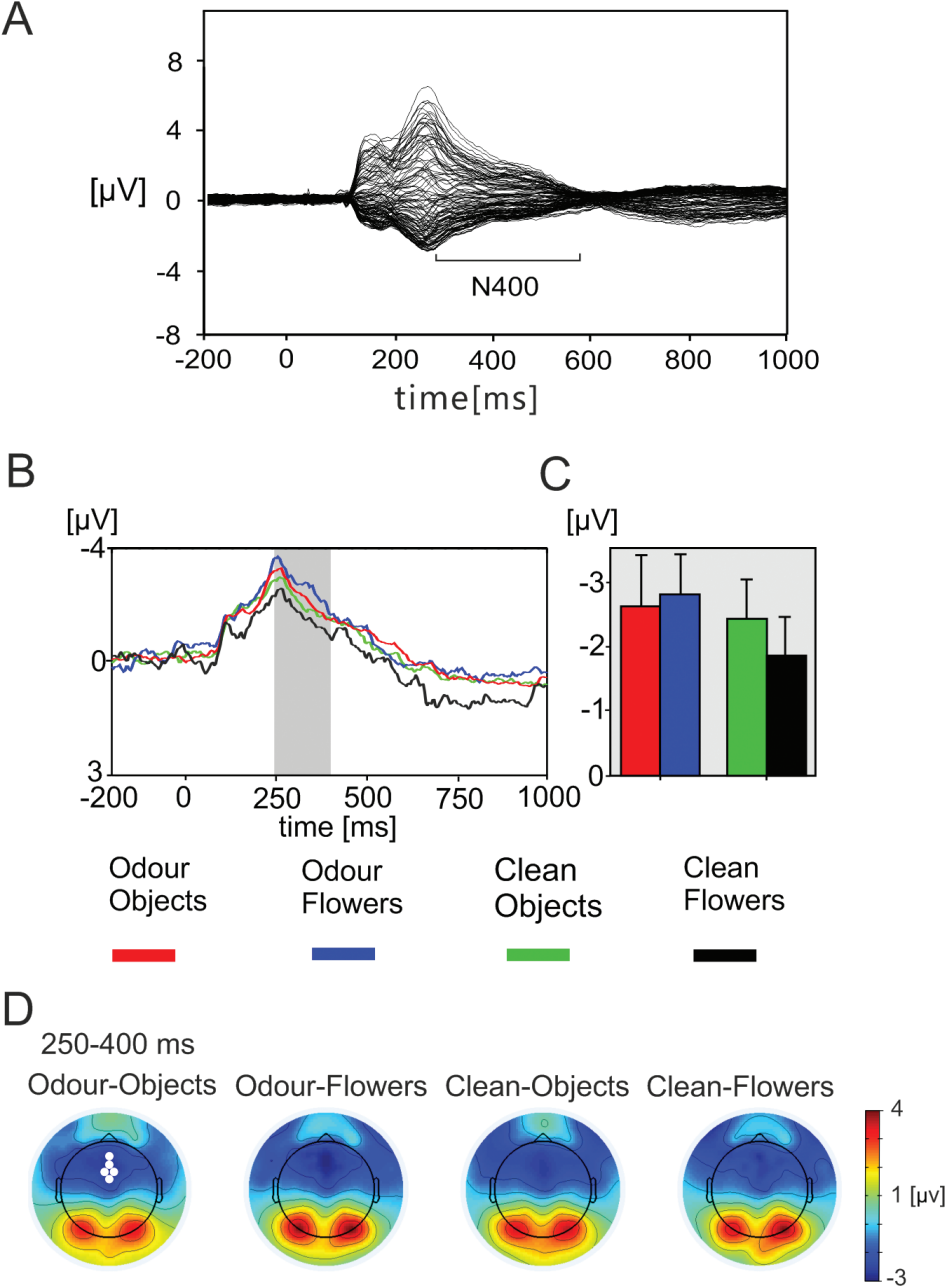
(A) The butterfly plot of grand averaged data from all odor and picture conditions representing the ERP associated with the onset of picture stimuli from all electrodes. The period of N400 negativity established from previous studies is highlighted. **(B)** Mean event-related potential for each picture type and odor condition in the early period of odor/clean presentations. The ERPs represent the average data from the cluster of electrodes identified by omnibus analysis (white circles panel D) and the gray rectangle indicates the period demonstrating a significant interaction between odor condition and picture type. Red = odor condition with incongruent object pictures; blue = odor and congruent flower pictures; green = clean air with object pictures; black = clean air condition with flower pictures. **(C)** Bar chart illustrating the mean amplitude and standard error bars for the N400 component (250–400 ms, gray rectangle, panel B) from select electrodes. **(D)** Scalp isopotential maps demonstrating the topography of the ERP for each condition during the period 250–400 ms after picture onset.

To investigate the effects of odor–visual congruence across the period of exposure, EEG amplitudes from a cluster of fronto-central electrodes in the period 250–400 ms after picture onset (which demonstrated a main effect of prolonged odor presentation time in the localizer) were investigated. Visual inspection of ERPs and topographic maps for this period displayed a consistent negative potential component over frontal-central regions corresponding to N400. Figure 5 shows the butterfly plot of the grand average ERP data encompassing both odor and picture congruence conditions. N400 amplitudes corresponding to this time period and electrode cluster were exported for each individual participant for analysis utilizing 2 × 2 within-subjects ANOVAs (odor × congruence) for each of the 5 time windows.

A 2 × 2 (odor × picture type) within-subjects ANOVA for the first time window (when odor perception was strongest) revealed a main effect of odor, with stronger frontal negativity from 250 to 400 ms in odor blocks relative to clean air blocks (*F*(1,24) = 6.34, *P* = 0.019). There was no significant effect of picture type for trials from the first (early exposure) time window, but the interaction was significant (*F*(1,24) = 7.37, *P =* 0.012), indicating a difference in the effect congruent, relative to incongruent, pictures depending on the presence of odor. Paired samples *t*-tests indicated greater N400 negativity in odor trials with congruent images (*t*(24) = −2.94, *P* = 0.007), which was not evident in clean air trials (*t*(24) = −1.36, *P* = 0.19). Figure 5 shows the average ERP curves from significant electrodes and bar charts illustrating mean amplitudes for each group and condition in the first time window; scalp isopotential maps of ERP components for each group and picture type are shown averaged across the N400 time window. ANOVAs for the subsequent time windows covering the remainder of the prolonged odor exposure revealed no significant main effects of odor/picture type or significant interactions.

### Distributed source analysis

**Figure 6.**
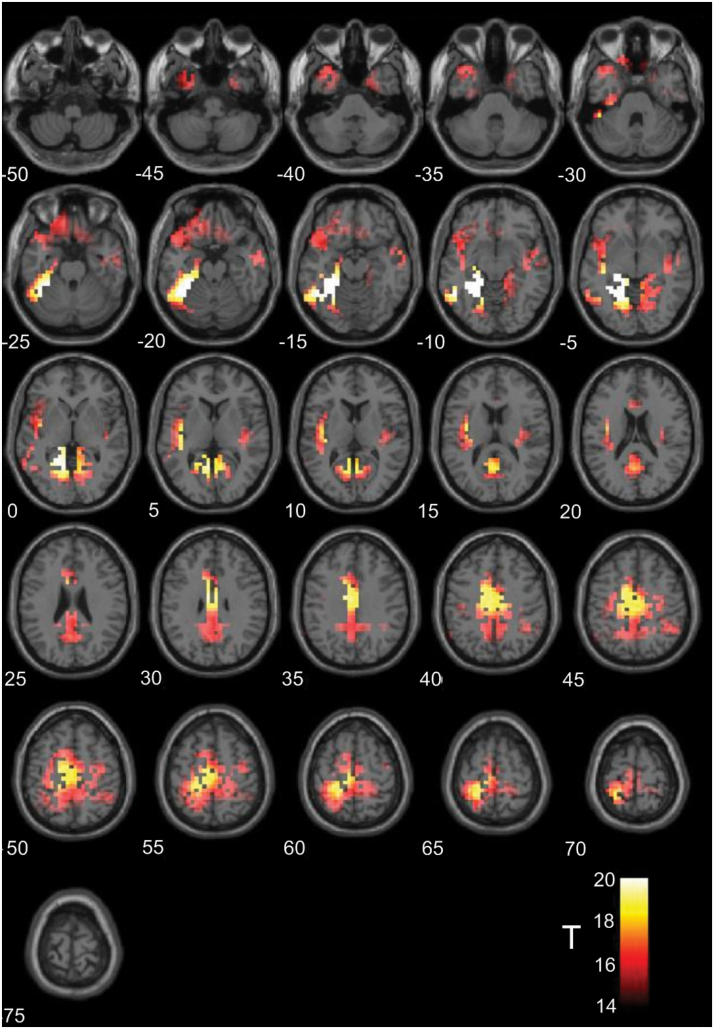
Axial montage illustrating the peaks of source activation throughout the whole brain identified by univariate analyses (*T* > 15) of grand average data in the early time window (which demonstrated an interaction between odor and picture type in scalp analyses).

The sLORETA output for peak source generators of topographic ERPs in the N400 time window (250–400 ms) is illustrated in Figure 6. The activations represent the grand average of activity for all conditions in the first time window of odor exposures. Univariate analysis of sLORETA maps revealed 12 distinct clusters of activation. These regions included bilateral orbitofrontal cortices; right inferior frontal gyrus; bilateral insula cortices; anterior, mid, and posterior cingulate cortex, bilateral parahippocampal gyri; bilateral lingual gyri; and right parietal cortex. These regions represent the peak sources of activation during the N400 component period for all conditions in the first period of odor and clean air blocks. We exported sLORETA values from each of these sources and for each condition using a spherical (10 mm diameter) ROI centered on the peak value of each cluster. The values for each ROI were utilized in a 2 × 2 (odor × picture type) within-subjects ANOVA but no regions exhibited any significant interaction effect that survived correction for multiple comparisons.

## Discussion

The findings of study 1 revealed an effect of odor–visual congruence for improved odor detection sensitivity, which persisted throughout the period of exposure. However, odor detection sensitivity diminished over time, and the cubic interaction between odor and picture type points to a fading influence of odor–visual congruence as habituation occurs. This supports our first hypothesis. RT data for accurate odor detections in the congruent condition were shorter throughout the entire exposure, pointing to an influence of odor–visual congruence on central processing, but there was no evidence of any difference as the exposure progressed. This demonstrates that congruent visual cues did not significantly reduce the degree or scale of habituation, although they do improve detection sensitivity consistently across the whole exposure period. Study 2 expanded on the previous study to elicit the understanding of how central processing of odor–visual perception may be affected by the influence of congruence throughout habituation to prolonged exposure. ERPs relating to the onset of picture presentation in the presence or absence of odor point to changes in electrophysiological processing of images over the period of exposure. A significant interaction effect (i.e., an influence of odor–visual congruency) was evident in the N400 frontal negativity during the early period of odor exposure when perception was at its strongest level. During this early period when odor perception was strongest, congruent odor–visual pairs elicited the strongest N400 negativity. Source analysis of the N400 component from trials in the early time window revealed a complex array of active sources relating to the scalp data.

Our behavioral findings revealed that congruent odor–visual pairs resulted in a shorter response time and improved sensitivity, which corresponds with previous research (Gottfried and Dolan 2003). However, we have expanded on this by demonstrating that, although congruence affects performance throughout the entire prolonged exposure period, habituation diminishes odor perception sensitivity and the influence of visual congruence. Previous studies reported enhanced frontal negativity with odor, relative to clean air, in an odor–visual pair paradigm (Lorig et al. 1993, 1995), which accords with our own findings and which may suggest that frontal negativity is indicative of the influence of odor primes on subsequent visual processing. However, several previous studies have indicated that incongruent odor–visual pairs may be accompanied by enhanced N400 negativity (Grigor 1995, 1999; Castle et al. 2000), which diverges from the congruence effect seen in the present study. However, these studies all utilized a minority (25%) of rare *incongruent* trials (opposite to the balance in the present study) and also requested explicit response to categorize stimuli as congruent or incongruent (not odor detection). In the present study, enhanced N400 could stem from the fact that “congruent” odor–picture pairs are a rare event in the current paradigm (at a frequency of 25%) and, therefore, represent an expectation violation that could result in enhanced frontal negativity. Our findings suggest that the N400 may encode aspects of the salience of the stimuli, which can be boosted by either congruence or incongruence depending on rarity or context. This interpretation would be in agreement with the opinion that the N400 response may pertain to stimuli that violate a previously established context (Pratarelli 1994). An electrophysiological review also concluded that N400 represents a signature of complex processing encompassing aspects of perception, attention, memory, and semantics, which combine to influence the manner in which we infuse our environment with meaning (Kutas and Federmeier 2011).

In light of the literature, it would be overly simplistic to infer that the N400 component in response to odor–visual pairs relates directly and solely to congruence, and this may explain why some previous cross-modal studies failed to elicit N400 differences (Bensafi et al. 2002; Robinson et al. 2015). Instead, we can view our findings, and previous research, as indicative that the N400 represents a valid research target for aspects of odor–visual processing that impact on the interpretation of our environment. However, further research is required to fully elucidate the mechanisms by which this modulates perception. For example, in the present study, we are limited by the fact that congruence effects cannot be dissociated from the effects of the rarity of stimuli. Furthermore, different odors offer different profiles of habituation (Sinding et al. 2017), which may also play a role in subsequent interaction with visual cues. In future, it is possible that this experimental paradigm can be expanded to better facilitate the N400 as a research target, for example, by using a wider range of odor–visual congruence pairings to better elucidate congruence (Sarfarazi et al. 1999) or by refining the paradigm to incorporate participant feedback in the form of categorizing the congruency of each pairing, which was shown to improve N400 modulation in relation to the context in previous studies.

One previous study utilized odor–visual pairs with an exposure time of 1 min, but the researchers did not analyze the effect of time on the central processing of odor–visual pairs (Sarfarazi et al. 1999). Therefore, our finding of N400 effects diminishing as odor exposure progresses gives the first indication that this component could be modulated by ongoing habituation to odor. Source localization of the significant congruence effect in N400 points to a complex array of cortical sources, but, perhaps, of most relevance is the inclusion of bilateral orbitofrontal, insula, and parahippocampal sources and cingulate sources during the N400 processing time period. Occipital sources in bilateral lingual gyri are most likely related to concurrent visual processing. Parahippocampal regions and orbitofrontal cortex were previously highlighted as regions with importance for the integration of odor–visual input using fMRI (Gottfried and Dolan 2003), and positron emission tomography revealed an integrative role for insula activation, which was only present in cross-modal olfactory–gustatory processing (Small et al. 1997). Previously, the conditioning of congruent odor–visual stimuli was shown to evoke olfactory-like activation in orbitofrontal, insula, hippocampal, and cingulate cortices for subsequent visual stimuli (Karunanayaka et al. 2015). Therefore, these regions are likely to be important for the interaction of odor–visual processing, which contributes to a holistic percept in the human brain.

To conclude, together our studies demonstrate an interaction between the congruence of cross-modal odor–visual pairs and the ongoing process of habituation to odor. This highlights the existence of a relationship between top-down psychological factors and the habituation process. Our previous research highlighted the influence of attention on habituation (Fallon et al. 2018), but the present findings begin to shed light on the impact of habituation on central-cognitive cross-modal processing. The findings support the bidirectional relationship between odor–visual processing; congruent visual cues influence behavioral measures of odor perception, and odor also affects electrophysiological processing of visual cues. For the first time, we show evidence of a shift in the relationship as odors prolong and habituation occurs. Our findings also indicate support for the N400 component as a potential marker of the influence of context and congruence during odor habituation with cross-modal visual stimuli.
